# Analysis of the expression and localisation of a LAP protein, human scribble, in the normal and neoplastic epithelium of uterine cervix

**DOI:** 10.1038/sj.bjc.6601465

**Published:** 2004-01-06

**Authors:** S Nakagawa, T Yano, K Nakagawa, S Takizawa, Y Suzuki, T Yasugi, J M Huibregtse, Y Taketani

**Affiliations:** 1 Department of Obstetrics and Gynecology, Graduate school of Medicine, University of Tokyo, Hongo 7-3-1 Bunkyo-ku, Tokyo 113-8655, Japan; 2 Department of Radiology, Graduate school of Medicine, University of Tokyo, Tokyo 113-8655, Japan; 3 Institute for Cellular and Molecular Biology, Section of Molecular Genetics and Microbiology, University of Texas at Austin, Austin, TX 78712, USA

**Keywords:** human scribble, LAP protein, cervical cancer, intracellular localisation

## Abstract

Recently, a LAP protein, scribble, was identified in *Drosophila* epithelia as a basolateral protein that controls the apical-basolateral polarity. Loss of scribble causes disorganisation and overgrowth of the epithelia. Scribble has a human homologue, human scribble (hScrib), which is a substrate of ubiquitin-mediated degradation by human papillomavirus E6 and the E6AP ubiquitin-protein ligase. In the present study, we revealed that hScrib localised to the basolateral regions of the epithelial cell line MDCK and human uterine cervical epithelial tissues by immunofluorescence. Human scribble colocalised rather with the adherens junction protein E-cadherin, but not with the tight junction protein ZO-1. Histochemical analysis showed a dramatic decrease in the expression of hScrib with the progression of disease from normal uterine cervical tissues to invasive cervical cancers through the precursor lesions. In contrast, the expression of hScrib was retained in the throughout epithelial layer of the HPV-negative cervical high-grade squamous intraepithelial lesions (H-SIL). Although quantitative RT–PCR revealed no significant downregulation of hScrib mRNA expression in the H-SIL, it revealed a clear downregulation in the invasive cancers. These results suggest the possibility that degradation by HPV E6 is one of the causal roles for the progressive decrease of hScrib expression during the disease progression from low-grade squamous intraepithelial lesions to H-SIL, and a cooperative role of downregulation of hScrib mRNA expression and ubiquitin-mediated degradation of hScrib by E6 and E6AP led to the complete decrease of hScrib expression during the process of carcinogenesis from H-SIL to invasive cancer. These data underscore the importance of hScrib in the construction of tissue architecture and prevention of cancer development.

The organised polarity of cells is a hallmark of the epithelia. In cancer cells, the apical-basolateral polarity is disrupted and mislocalisation of many junctional proteins or hormone receptors is reported. Recently, Bilder *et al* (Bilder and Perrimon, 2000) identified the basolateral protein scribble that determines the localisation of the apical epithelial determinants. Loss of scribble mutation leads to loss of apical-basolateral polarity and the apical determinant Crumbs shows unrestricted localisation not just at the apical region in *scribble* mutants. scribble is localised at the septate junction, which is the analogue of the vertebrate tight junction. In *scribble* mutants, the adherens junctions are misdistributed around the cell periphery (Bilder and Perrimon, 2000). How do the basolateral proteins determine the localisation of the apical determinants at the apical region of cells and the precise localisation of the adherens junctions just above the septate junctions? Looking at its amino-acid sequence, scribble has four PDZ domains (named for its occurrence in the PSD-95, Discs-Large, and ZO-1 proteins), and it could have four different binding partners through four PDZ domains simultaneously. In addition, scribble has 16 canonical leucine-rich repeats (LRRs), another protein–protein interaction motif. Related repeats to the LRRs are known to bind small GTPases of the Ras superfamily (Jou and Nelson, 1998; Kaibuchi *et al*, 1999). Recently, proteins with both PDZ domains and LRRs have been reported to have common functions in the control of the cell shape and polarity. These proteins are grouped as LAP (LRR and PDZ domain) proteins (Bilder *et al*, 2000a). Scribble is supposed to function as a scaffold protein using these protein-binding motifs. Loss of scribble mutation also leads to the overgrowth of imaginal discs, follicle epithelia, and larval brain (Bilder *et al*, 2000b). Controlling the cell apical-basolateral polarity is closely related to the growth control (Greaves, 2000). So, scribble is not only an apical-basolateral polarity determinant but also a tumour suppressor.

*Drosophila* scribble has a human homologue, human scribble (hScrib), which was identified as a substrate of ubiquitin-mediated degradation by high-risk human papillomavirus (HPV) E6 and the E6AP ubiquitin-protein ligase (Nakagawa and Huibregtse, 2000). Tumour suppressor p53 is targeted for ubiquitin-mediated degradation by E6 dependent on the E6AP (Huibregtse *et al*, 1993; Scheffner *et al*, 1993, 1994; Huibregtse *et al*, 1995). P53 had a key role in the control of cell cycle and apoptosis (Kuerbitz *et al*, 1992; Lowe *et al*, 1993). By degrading p53, E6 disrupts the self-defending machinery against DNA damages of the host cells. Recently, it has been shown that the E6 targets not only the nuclear substrate but also the cytoplasmic substrates such as hScrib via ubiquitin-mediated pathway (Kiyono *et al*, 1997; Nakagawa and Huibregtse, 2000; Mantovani *et al*, 2001). The degradation of hScrib by E6 and E6AP was shown in the *in vitro* setting (Nakagawa and Huibregtse, 2000).

We sought to address two issues in this study. The first issue is whether hScrib shows basolateral localisation analogous to that of *Drosophila* scribble. The Drosophila scribble localises at the septate junction, which is functionally analogous to the vertebrate tight junction. We analysed localisation of hScrib on the subject of the localisation of other junctional proteins in the MDCK cell line and primary human cervical epithelia. Carcinogenesis of the uterine cervix has multistages from normal cervical epithelia to the invasive carcinoma through the squamous intraepithelial lesions. The second issue that we sought to address is whether hScrib is involved in the carcinogenesis of the uterine cervix. If so, the localisation of hScrib could be disrupted in cancer tissues and its expression is supposed to decrease with the progression of diseases, possibly depending on the E6 and E6AP. These issues would be discussed in the present study.

## MATERIALS AND METHODS

### Construction of the anti-hScrib antibodies

The DNA sequence, which encodes C-terminus (amino acids 1208–1632), was subcloned into the pGEX-6P-1 vector (Amersham Pharmacia, Buckinghamshire, UK). Glutathione S-transferase (GST) fusion protein, GST-hScrib C-terminus, was made in the bacteria and purified according to the manufacturer's recommendation. The amino acids encoding hScrib C-terminus were cleaved from the GST-fusion proteins with the PreScission Protease (Amersham Pharmacia, Buckinghamshire, UK) and purified. This hScrib C-terminus was injected into rabbits as antigens. The hScrib C-terminus antibody (anti-hScrib C-terminus) was purified from the serum of the immunised rabbits by affinity chromatography.

### Western blotting

293 T cells were grown in DMEM medium supplemented with 10% foetal bovine serum. Protein extracts of 293 T cells, primary cervical tissues, and normal control tissues were made in the NP-40 lysis buffer containing 100 mM Tris (pH 8.0), 100 mM NaCl, and 1% NP-40. For primary tissues, cryostat sections were cut for haematoxylin and eosin staining to confirm the spread of the neoplastic lesions, and non-neoplastic tissues were trimmed off from the frozen tissues. For the normal cervix samples, we also trimmed off the underlying connective tissues. Protein concentration was determined by standard Bradford assay. Equal amounts of extracts were fractionated by SDS–PAGE and electrophoretically transferred onto the polyvinylidene difluoride membranes (Millipore Co., Bedford, MA, USA). The anti-hScrib C-terminus antibody (anti-hScrib C-terminus) was used at the dilution of 1 : 1000 to detect the expression of hScrib as indicated. The *in vitro*-translated hScrib and human homologue of *Drosophila* Discs-Large using the reticulocyte lysate system (Promega Co., Madison, WI, USA) were used as the positive and negative control, respectively. The level of protein expression was analysed by the STORM 860 according to the manufacturer's recommendation (Molecular Dynamics).

### Immunofluorescence of MDCK cells

Subconfluent MDCK cells were grown on coverslips in the culture medium. Cells were washed three times with phosphate-buffered saline (PBS) and then fixed with 3.7% formaldehyde in PBS for 10 min. Cells were then washed three times with PBS, rinsed with distilled water, and permeabilised with acetone at −20°C for 10 min. Cells were washed with PBS and incubated with the diluted anti-hScrib C-terminus and anti-ZO-1 antibodies (ZYMED, San Francisco, CA, USA) to stain the tight junctions, or anti-E-cadherin (BD Trasnduction laboratories, Lexington, KY, USA) to stain the adherens junctions for 30 min at room temperature. Cells were then washed three times with PBS and incubated with rhodamine or FITC-conjugated secondary antibodies (Sigma, St Louis, MO, USA), and then washed three times with PBS. Cells were mounted on a slide glass and examined by confocal fluorescence microscopy (Zeiss LSM 410 microscope). Images were captured with a CCD camera.

### Detection and typing of HPV DNA by PCR with consensus primers in the L1 ORF

The presence and type of HPV were determined by a PCR-based assay (L1-PCR), originally described by Yoshikawa *et al* (1991) and modified by Nakagawa *et al* (1999). The L1 region was amplified in 40 PCR cycles of 1.5 min at 95°C, 1.5 min at 48°C, and 2 min at 70°C, using consensus L1 primers L1C1 (5′-CGTAAACGTTTTCCCTATTTTTTT-3′, 1 M), L1C2 (5′-TACCCTAAATACTCTGTATTG-3′, 0.5 M), and L1C2M (5′-TACCCTAAATACCCTATATTG-3′, 0.5 M). Each reaction product (10 l) was electrophoresed on a 4% agarose gel, stained with ethidium bromide, and viewed under UV light. HPV types were identified based on restriction fragment length polymorphism (RFLP). The initial typing of amplified HPV DNA was carried out by the double digestion with *Dde*I and *Rsa*I, and then confirmed by digestion with at least three enzymes selected from *Acc*I, *Alu*I, *Bst*XI, *Fok*I, *Hae*III, *Hinf*I, *Kpn*I, *Mae*I, *Mae*III, *Pst*I, and *Xba*I. This L1-PCR can type at least 25 genital HPVs (type 6, 11, 16, 18, 30, 31, 33–35, 42–45, 51–56, 58, 59, 61, 66, 68, and 70).

### Immunohistochemistry and immunofluorescence of uterine cervical tissues

Normal cervical tissues and cervical neoplastic tissues were taken in pairs from patients with cervical cancer or its precursor lesions who gave written informed consent. The cervical tissues, which were used in this study, are as follows: normal cervix: 12 cases, cervical condyloma: five cases, low-grade squamous intraepithelial lesions (L-SIL): six cases, high-grade squamous intraepithelial lesions (H-SIL): 12 cases, and invasive cervical cancers: 16 cases. All the condylomas and the cervical neoplasm were positive for low-risk HPV 6/11 and high-risk HPV 16/18, respectively, except one HPV-negative case with the H-SIL. The tissue blocks were embedded in Tissue-Tek OCT compound and quickly frozen in dry ice acetone. Frozen sections (4 *μ*m) were taken on silane-coated glass slides. Sections were fixed in cold acetone for 10 min at −20°C and then rinsed in PBS. Slides were incubated with 10% normal pig serum for 20 min at room temperature to block nonspecific staining and then incubated with the anti-hScrib C-terminus antibody for 60 min at 37°C. Slides were rinsed in PBS and then incubated with horseradish peroxidase (HRP)-conjugated goat anti-rabbit IgG (H+L chain) (Medical and Biological Laboratories, Nagoya, Japan) as the second antibody for 60 min at 37°C. After washing in PBS, the sites of HRP were visualised by 3,3′-diaminobenzidine tetrahydrochloride and 30% hydrogen peroxide in PBS at room temperature. Slides were washed in distilled water and counterstained with 5% methyl green for 15 min. Slides were washed in distilled water and dehydrated through alcohol and xylene, and then mounted with a coverslip.

The immunohistochemical staining patterns of hScrib were compared between the normal epithelium and cervical neoplasm. We also analysed the precise localisation of hScrib in normal endocervical tissues in connection with the junctional proteins by immunofluorescence.

### Quantitative RT–PCR analysis of the hScrib mRNA

Total RNA was extracted from all the tumour specimens and the corresponding normal cervical tissues using the RNeasy Mini Kit (Qiagen, Inc., Chartsworth, CA, USA). From 20 *μ*g of total RNA, mRNA was isolated by the Oligotex-dT Super (TaKaRa Biomedicals, Japan). For the RT–PCR, the total volume (10 *μ*l) of mRNA preheated at 65°C for 5 min served as a template for single-strand cDNA synthesis in a 20 *μ*l reaction mixture containing 3 mM MgCl_2_, 75 mM KCl, 50 mM Tris-HCl (pH 8.3), 0.5 mM dNTPs, 200 *μ*M oligo (dT) primer, 20 U of RNase inhibitor, and 200 U of M-MLV reverse transcriptase (Gibco BRL, Gaithersburg, MD, USA) at 37°C for 60 min. The reaction was terminated at 95°C for 3 min. To ensure the fidelity of mRNA extraction and RT reaction, all samples was subjected to PCR amplification with oligonucleotide primers specific for the constitutively expressed housekeeping gene GAPDH. At least three different PCR reactions were performed using the LightCycler-FastStart DNA Master SYBR Green I kit (Roche) for each sample. The expression level of hScrib mRNA was standardised as a ratio to the level of GAPDH mRNA. The primer sequences for hScrib were as follows: forward, 5′-GGSTCCATGCTCAAGTGCATCCCGC-3′ and reverse, 5′-GCGGCCGCTTAGAAGTTGGCCACCT-3′. PCR amplifying condition started at 95°C for 10 min, followed by 37 cycles of 95°C for 15 s, 67°C for 10 s, and 72°C for 18 s.

## RESULTS

### Construction of the anti-hScrib antibody

**Figure 1:**
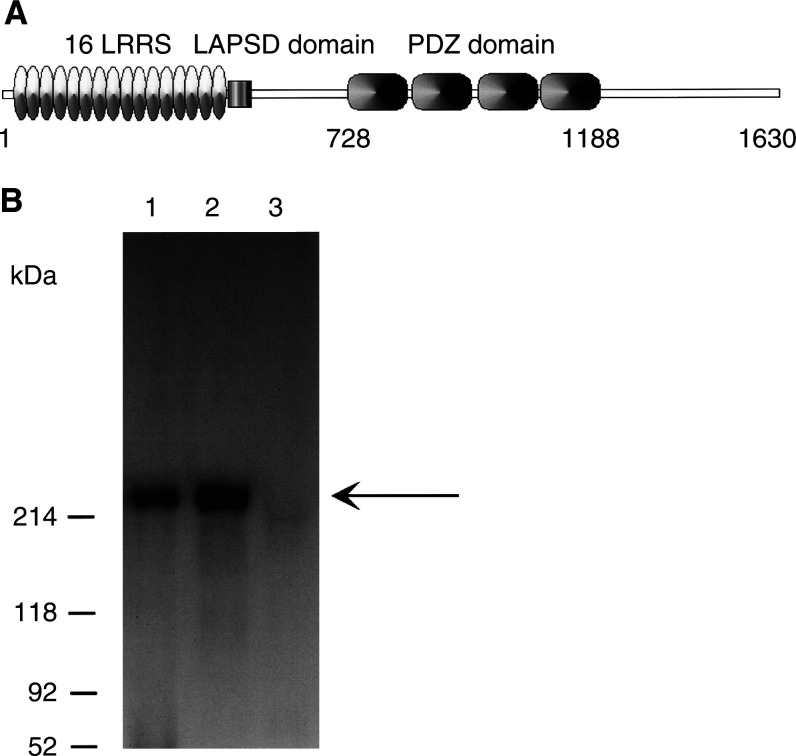
Construction of the anti-hScrib antibody using its C-terminus. (**A**) Structure of hScrib consists of 16 canonical LRRs, followed by LAPSD (LAP-specific domain) and four PDZ domains. We generated an anti-hScrib antibody (anti-hScrib C-terminus) using C-terminus (AA 1208–1630) as antigens. (**B**) Anti-hScrib C-terminus antibody detected the endogenous hScrib protein with 220 kDa in molecular weight in 293 T cells (lane 1) and *in vitro*-translated hScrib (lane 2), but not *in vitro*-translated hDlg by Western blotting (lane 3).

To generate anti-hScrib antibody, a synthetic peptide sequence of the C-terminus (amino acids 1208–1632) of hScrib was selected (Figure 1A

). With the prepared anti-hScrib C-terminus antibody, we analysed the extracts of 293 T cells by Western blot analysis to test whether the anti-hScrib antibody can detect the hScrib protein specifically. The anti-hScrib antibody detected a sharp band around 220 kDa, which corresponds to the *in vitro*-translated hScrib (Figure 1B). In contrast, it did not react to the *in vitro*-translated human homologue of *Drosophila* Discs-Large (hDlg), another PDZ domain containing protein. We concluded that the anti-hScrib antibody detects the hScrib protein specifically.

### hScrib localises at the basolateral membrane in the epithelial cell line

**Figure 2:**
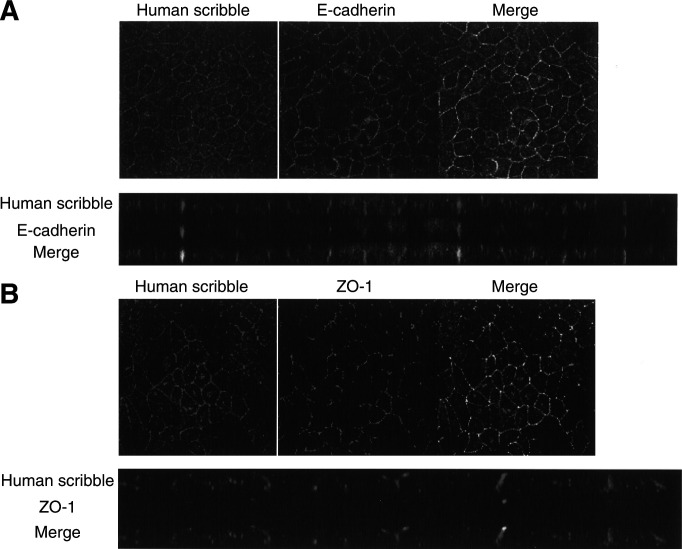
Confocal images of immunofluorescence staining of hScrib, E-cadherin, and ZO-1 in the MDCK epithelial cell line. (**A**) Partial colocalisation of hScrib and E-cadherin at the basolateral membrane. (**B**) Localisation of hScrib and ZO-1. ZO-1 localised at the top of the basolateral membrane, where hScrib did not localise.

*Drosophila* scribble localises at the septate junction, the equivalent of the mammalian tight junction. To test whether the endogenous hScrib localises at the basolateral membrane, especially at the tight junction, we analysed its localisation in the polarised canine epithelial cell line MDCK. Looking at the cross-section of the fluorescence image under a confocal microscope, the endogenous hScrib localised at the broad basolateral membrane (Figure 2A and B

). These data indicate the conserved homology of amino-acid sequence of scribble among the species. HScrib colocalised with the adherens junctional protein, E-cadherin, at most parts of the basolateral wall excluding the top of the adherens junction (Figure 2A), but not with ZO-1 (Figure 2B). HScrib localised just below the tight junction.

### hScrib is a basolateral membrane-associated protein in primary human tissues

**Figure 3:**
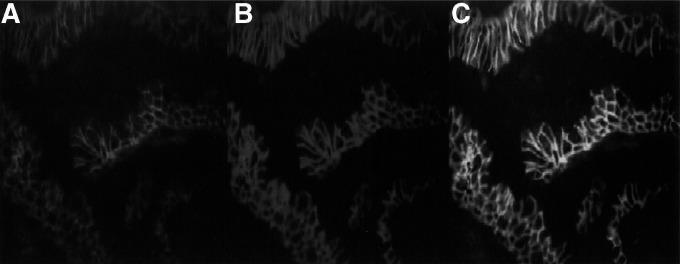
Immunofluorescence staining of hScrib and E-cadherin in uterine endocervical tissues. (**A**) Basolateral localisation of hScrib. (**B**) Localisation of E-cadherin, which shows the area of adherens junction. (**C**) Colocalisation of hScrib with E-cadherin, an adherens junctional protein.

To examine whether hScrib shows the basolateral localisation in primary human tissue, as it showed in the epithelial cell line MDCK, we analysed its localisation in uterine cervical tissues. HScrib localised at the cell–cell boundaries in the normal cervical epithelia. In the columnar endocervical tissues, the immunofluorescence analysis revealed the typically basolateral localisation of hScrib (Figure 3A

) and the colocalisation between hScrib and E-cadherin (Figure 3B), which is identical to the observation in cultured MDCK cells (Figure 2A).

### hScrib shows a dramatic decrease in the expression during the progression of disease

**Figure 4:**
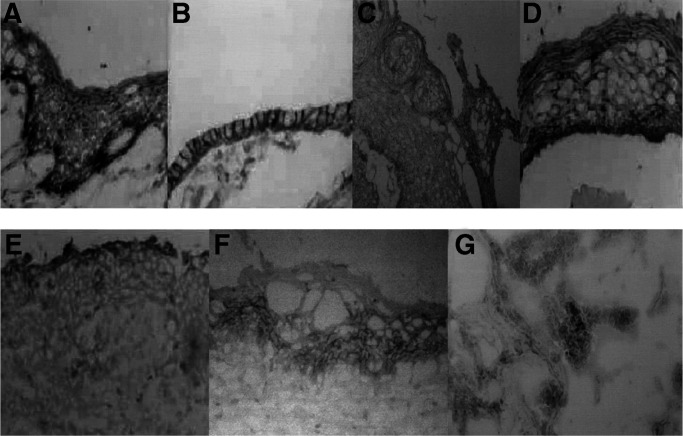
Immunohistochemical detection of hScrib in normal uterine cervical epithelia and neoplasm. There was a dramatic decrease in the expression of hScrib with disease progression. (**A**) Normal squamous epithelia. (**B**) Endocervical epithelia. (**C**) Cervical condyloma, an HPV-related wart. (**D**) L-SIL (CIN1). (**E**) H-SIL (CIN3). (**F**) Invasive cervical cancer.

**Figure 5:**
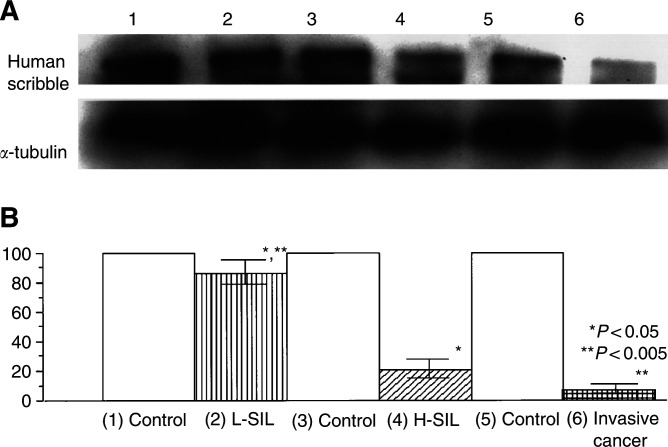
Western blot analysis of hScrib protein levels in cervical neoplasm and the normal counterparts using the anti-hScrib C-terminus antibody. (**A**) The representative result of three independent experiments. (**B**) Analysis of hScrib protein expression relative to *α*-tubulin in the cervical neoplasm. The results are expressed as the mean percentage of the normal counterpart. Lanes 1, 3, and 5: the normal counterpart; Lane 2: L-SIL; Lane 4: H-SIL; Lane 6: invasive cancer.

**Figure 6:**
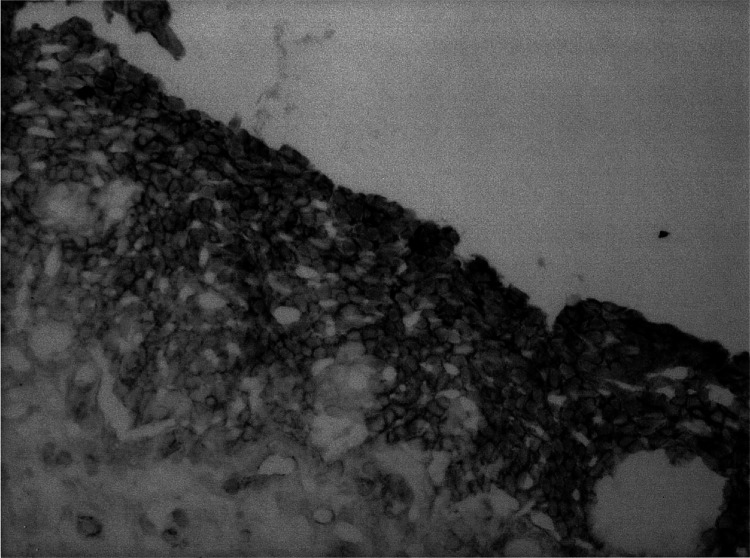
Immunohistochemical detection of hScrib in a case with H-SIL without HPV infection. The normal hScrib expression is seen throughout the epithelial layer.

To determine if hScrib is involved in the cervical carcinogenesis, we analysed its expression and localisation in cervical cancer and its precursor lesions (Figure 4

). The expression and localisation of hScrib in the cases of L-SIL was indistinguishable from that in the normal cervical epithelium (Figure 4A and D). In contrast to the L-SIL, hScrib showed a dramatic decrease in its expression in the cases of H-SIL (Figure 4E). There was a faint expression of hScrib only in the upper layer of the epithelia of H-SIL (Figure 4E). In contrast to hScrib, the expression of the adherens junction protein E-cadherin was retained along the basolateral membrane in the same case (data not shown). In the normal counterpart tissues, normal expression and localisation of hScrib was confirmed (Figure 4A and B). Human scribble showed the cell–cell boundary-associated localisation in the cases of cervical condyloma, which is a low-risk HPV-related wart (Figure 4C). In the invasive cervical cancer tissues, hScrib showed misdistributed localisation and fully decreased expression comparing its localisation and expression in the normal counterpart tissue of the case (Figure 4F). The other PDZ-domain protein hDlg, which is the substrate of HPV E6 for ubiquitin-mediated degradation, also showed a dramatic decrease in its expression with progression of disease (SN and ST, unpublished data), as described by Watson *et al* (2002). We compared the expression level of hScrib protein by Western blotting between cervical neoplasm and normal cervical tissues to see whether hScrib actually shows a decrease in the expression compared with the normal counterparts during the progression of disease. Western blotting could not detect any difference between the neoplasm and its normal counterpart in the expression of hScrib in the cases of L-SIL (Figure 5

, lanes 1 and 2). In contrast, it showed a clear difference between the neoplasm and its counterpart in the cases of H-SIL and invasive cancer (Figure 5, lanes 3–6), confirming our data of immunohistochemistry. Although hScrib showed a dramatic decrease in its expression with disease progression in the HPV-positive cervical neoplasms, its expression was retained throughout the epithelial layer of the HPV-negative H-SIL case (Figure 6

). Next we examined whether the decreased expression of hScrib is due to the reduced transcription of hScrib gene. The mean hScrib/GAPDH mRNA level analysed by the quantitative RT–PCR in all cervical neoplastic tissues and normal control tissues did not show a significant difference in normal cervical tissue (1.40±0.15), L-SIL (1.42±0.21), and H-SIL (1.33±0.08), except for the invasive cancer (0.31±0.16). These data suggest the possibility that degradation of hScrib by E6 and E6AP could have a role in the progressive decrease of hScrib expression during the disease progression from L-SIL to H-SIL.

## DISCUSSION

*Drosophila* scribble was identified as an apical-basolateral polarity determinant in the epithelia (Bilder and Perrimon, 2000). Loss of scribble mutation leads to the overgrowth of follicular epithelia and imaginal disc in *Drosophila* (Bilder *et al*, 2000b). Loss of polarity and tissue architecture is a common feature of cancer tissues. We sought to observe whether the human homologue of scribble is involved in human carcinogenesis. We showed that hScrib is a substrate of high-risk HPVs and E6AP for ubiquitin-mediated degradation (Nakagawa and Huibregtse, 2000). These data let us to analyse the involvement of hScrib in the uterine cervical carcinogenesis. The multistage nature of cervical carcinogenesis is suitable to investigate its involvement in cancer development. Before addressing this issue, we analysed its localisation in cultured epithelial cells and cervical tissues to see whether hScrib shows the basolateral localisation. Human scribble localised at the broad basolateral membrane in the epithelial MDCK cell line and human endocervical tissues as expected. Its localisation is not restricted to the narrow tight junction, and it colocalised rather with E-cadherin, the adherens junction protein. *Drosophila* scribble shows the colocalisation with the septate junction marker Coracle and does not colocalise with the adherens junction marker Armadillo, the homologue of vertebrate *β*-catenin (Bilder *et al*, 2000b; Bilder and Perrimon, 2000). The arthropod septate junction is thought to be functionally, but not structurally, analogous to the vertebrate tight junction. *Drosophila* scribble and hScrib have the conserved amino-acid sequence, especially in LRRs and PDZ domains, which propose an identical function for these two homologues. Another PDZ-domain protein, *Drosophila* Discs-Large, localises at the septate junction, but its human homologue hDlg localises at the basolateral membrane, and not just at the tight junction (Ide *et al*, 1999). These data are in line with our data with respect to the localisations of scribble at the septate junction in *Drosophila* embryonic epithelia and those of hScrib at the broad basolateral wall in the mammalian epithelial cells. Recently, we showed that the green fluorescent protein (GFP)-hScrib colocalised with ZO-1, the component of the tight junction in MDCK cells (Nakagawa and Huibregtse, 2000). There can be several reasons why the endogenous localisation of hScrib is different from that of the GFP-hScrib. GFP could misdistribute its original localisation. It is also likely that the vertebrate junctions consist of the completely assembled protein complex and the exogenous protein could not join it.

The normal squamous epithelium consists of multilayers of epithelial cells. In the present study, hScrib showed the localisation at the extensive cell–cell boundaries in the squamous epithelium, in contrast to its basolateral localisation in the endocervical columnar epithelium. The multistage nature of the cervical carninogenesis enabled us to investigate whether hScrib is involved in this process (de Boer *et al*, 1999). The localisation of hScrib at the cell–cell boundaries and its expression in L-SILs, in which the upper two-third of the epithelium has undergone cytoplasmic differentiation and cells in the lower one-third lack normal differentiation and maturation, was indistinguishable from that in the normal squamous epithelium. In contrast, the expression of hScrib diminished with the progression of disease from L-SILs to invasive cancers. In H-SILs, which was determined by the abnormal changes involving more than lower two-thirds of the epithelium, the expression of hScrib was almost negative, only leaving a very faint expression in the uppermost layer. In invasive cancers, immunohistochemistry could detect the very weak signals of hScrib in the cytoplasm arbitrarily and no hScrib expression at the cell–cell boundaries, which reflects the disruption of cell–cell junctions and mislocalisation of junctional proteins in invasive cancers. Our data show the evidence of involvement of hScrib in the human cancer development for the first time. The dramatic change in the expression of hScrib suggests that its function as a tumour suppressor could be disrupted during the process of progression from L-SIL to H-SIL. No significant difference in the level of hScrib mRNA expression between L-SIL and H-SIL suggests the possibility that the progressive decrease in the expression of hScrib during the development of cervical cancer especially from L-SIL to H-SIL is caused by degradation depending on HPV E6 and E6AP. The retention of the expression of hScrib in the HPV-negative H-SIL case also supports this possibility (Figure 6). To confirm the involvement of HPV E6 and E6AP more precisely, we need to analyse more HPV-negative H-SIL cases and invasive cancers. A cooperative role of downregulation of hScrib mRNA expression and ubiquitin-mediated degradation of hScrib by E6 and E6AP might lead to the complete decrease of hScrib expression during the process of carcinogenesis from H-SIL to invasive cancer.

Our data indicate that hScrib is involved in human carcinogenesis. Most of the mutations in about 50 tumour suppressor genes identified in *Drosophila* give rise to only overproliferation. Only three genes, scribble, Dlg, and Lethal giant larvae (Lgl), are responsible for retaining tissue organisation (Greaves, 2000; Wodarz, 2000; Bilder *et al*, 2000b). Mutation of these three genes shares the phenotype of loss of tissue architecture and overgrowth in embryonic imaginal disc and follicular epithelia (Bilder *et al*, 2000b). These three genes are grouped together as malignant neoplastic tumour suppressors and act in concert to regulate cell proliferation and tissue organisation. The two PDZ-domain proteins, scribble and Dlg, are thought to bind transmembrane protein and form a protein complex as the scaffold at the septate junction. In contrast to the PDZ-domain proteins, Lgl participates in the determination of cell polarity by targeted secretion of membrane proteins. The yeast homologue of Lgl binds to t-SNARE proteins, which is essential for the cargo-carrying vesicle targeting (Lehman *et al*, 1999; Msch *et al*, 2002). These three tumour suppressors have the individual human homologue, hScrib, hDlg, and human homologue of *Drosophila* tumour suppressor lethal giant larvae. High-risk HPVs target hScrib and hDlg for ubiquitin-mediated degradation depending on the E6AP ubiquitin-protein ligase (Nakagawa and Huibregtse, 2000) (For hDlg, unpublished data of SN and JMH). E6 and E6AP could degrade these two scaffolding PDZ tumour suppressors at the basolateral membrane and disrupt the junctional protein assembly at the basolateral region.

Our data indicate the involvement of hScrib in the development of human cancer and shed light on the closely related mechanism between construction of tissue architecture and prevention of cancer development. Further investigation of the interaction among these three tumour suppressors in epithelial cells and cancer cells will give us more knowledge regarding carcinogenesis. These studies are warranted and ongoing in our laboratory.
